# Myelin oligodendrocyte glycoprotein antibody‐associated optic neuritis in a post‐COVID‐19 infection patient

**DOI:** 10.1002/iid3.1051

**Published:** 2023-10-25

**Authors:** Mohamad Azlan Zaini, Ayesha Mohd Zain, Norshamsiah Md Din, Chenshen Lam

**Affiliations:** ^1^ Department of Ophthalmology, Faculty of Medicine Universiti Kebangsaan Malaysia Kuala Lumpur Malaysia

## Abstract

**Purpose:**

SARS‐CoV‐2 viral infection affects multiple systems including the respiratory, gastrointestinal, neurological, cardiac, and ophthalmic systems. We report a case of myelin oligodendrocyte glycoprotein (MOG) related optic neuritis in a SARS‐CoV‐2 (COVID‐19) patient.

**Methods:**

Case report.

**Results:**

A 36‐year‐old Malay gentleman with underlying hypertension presented with the first episode of bilateral progressively worsening blurred vision for 1 week associated with retrobulbar pain. There were no other neurological symptoms. He had fever a week before the eye symptoms and tested positive for COVID‐19. He received COVID‐19 booster vaccine a month before the disease onset. On examination, his vision was hand motion on right eye and 6/18 on left eye. Relative afferent pupillary defect (RAPD) was positive on right eye with abnormal optic nerve function tests. Anterior segments were unremarkable. Fundus examination showed bilateral optic disc swelling. MRI revealed multifocal hyperintense subcortical white matter lesions. Optic nerves appeared normal with no enhancement seen. Blood investigation showed a positive serum MOG antibody. Intravenous methylprednisolone was commenced followed by oral prednisolone after which his vision and ocular symptoms markedly improved. The oral prednisolone was tapered alongside addition of azathioprine. At 1 month, the disease was stable with no recurrence.

**Conclusion:**

While optic neuritis has been associated with both COVID‐19 infection and vaccination, MOG IgG antibody‐mediated optic neuritis is also a possible manifestation. This type of optic neuritis associated with COVID‐19 infection does not show a similar pattern of frequent recurrences as seen in non‐COVID‐19 related optic neuritis.

## 1 INTRODUCTION

COVID‐19 has been found to cause a wide range of health implications affecting various organs such as the heart, kidney, nervous system, gastrointestinal tract, and eyes.^1^ In the nervous system, the virus has the ability to dysregulate the immune system, triggering sequential autoimmune events and leading to various neurological clinical manifestations.^2^, ^3^ In this report, we describe a case of a COVID‐19 positive young gentleman who developed bilateral optic neuritis associated with a positive myelin oligodendrocyte glycoprotein (MOG) IgG antibody.

## 2 CASE REPORT

A 36‐year‐old Malay gentleman with underlying hypertension presented with bilateral progressive blurring of vision for 1 week associated with retrobulbar pain. There was no recent headache or any other neurological symptoms. He had fever 1 week before the ocular symptoms in which he was tested positive for COVID‐19 infection through RTK antigen. However, he did not develop any respiratory tract symptoms, anosmia or ageusia. He also gave a history of receiving COVID‐19 booster vaccination 2 months before the disease onset. He was diagnosed with mild COVID‐19 infection and was self‐quarantined at home without the need for hospitalization.

**Figure 1:**
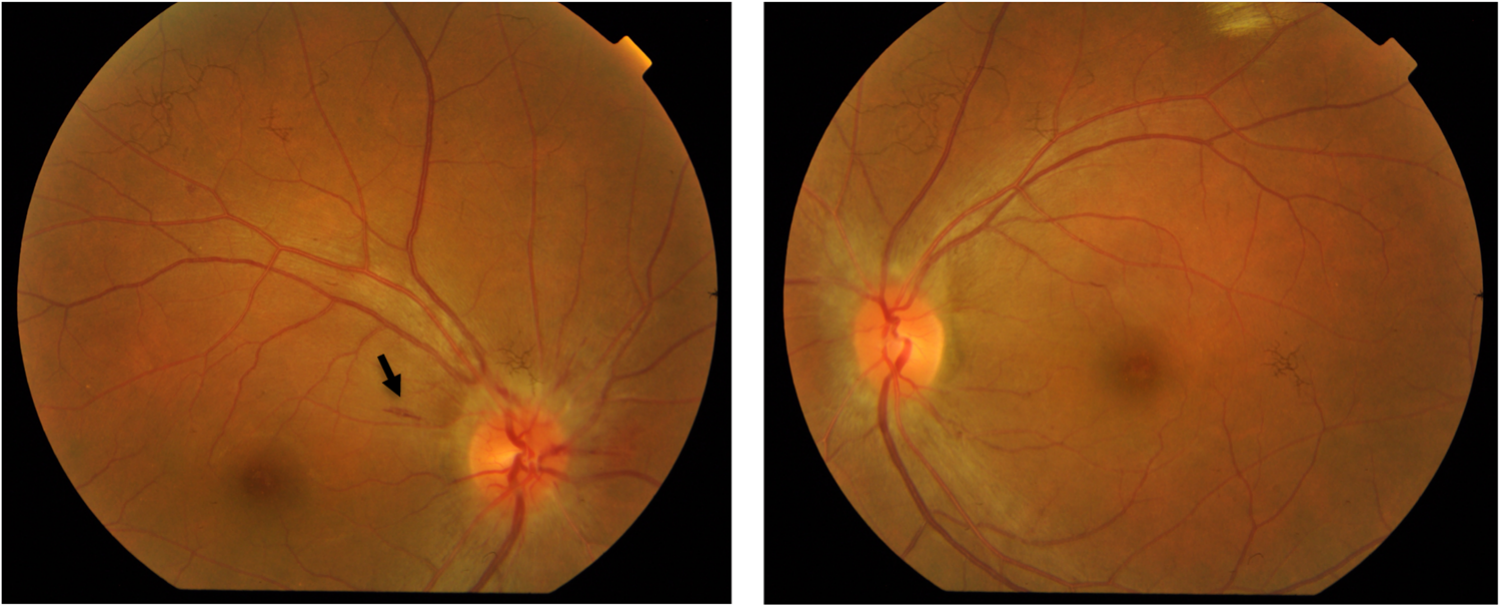
Bilateral optic disc swelling seen at presentation. Circumferential halo of disc edema and splinter haemorrhage (black arrow) temporally (black arrow) was seen on the right eye and C‐shaped halo of disc edema was seen sparing the temporal disc margin on left eye.

Examination revealed hand motion vision in the right eye and 6/18 in the left eye, with a relative afferent pupillary defect and optic nerve dysfunction on the right eye. The left eye had a normal optic nerve function. Other cranial nerve examinations were normal. Dilated fundus examination revealed bilateral disc oedema with splinter haemorrhages on the right eye (Figure 1). There were no cotton wool spots, macular stars, vasculitis, or other retinal changes seen.

**Figure 2:**
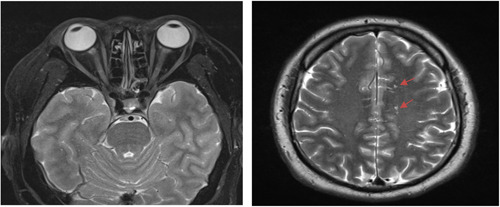
T2 weighted MRI of the brain and optic nerve showing no optic nerve enhancement. There are multifocal hyperintense white matter lesions at the subcortical regions of bilateral frontal lobes, left parietal lobe and left posterior cingulate gyrus (orange arrows).

**Figure 3:**
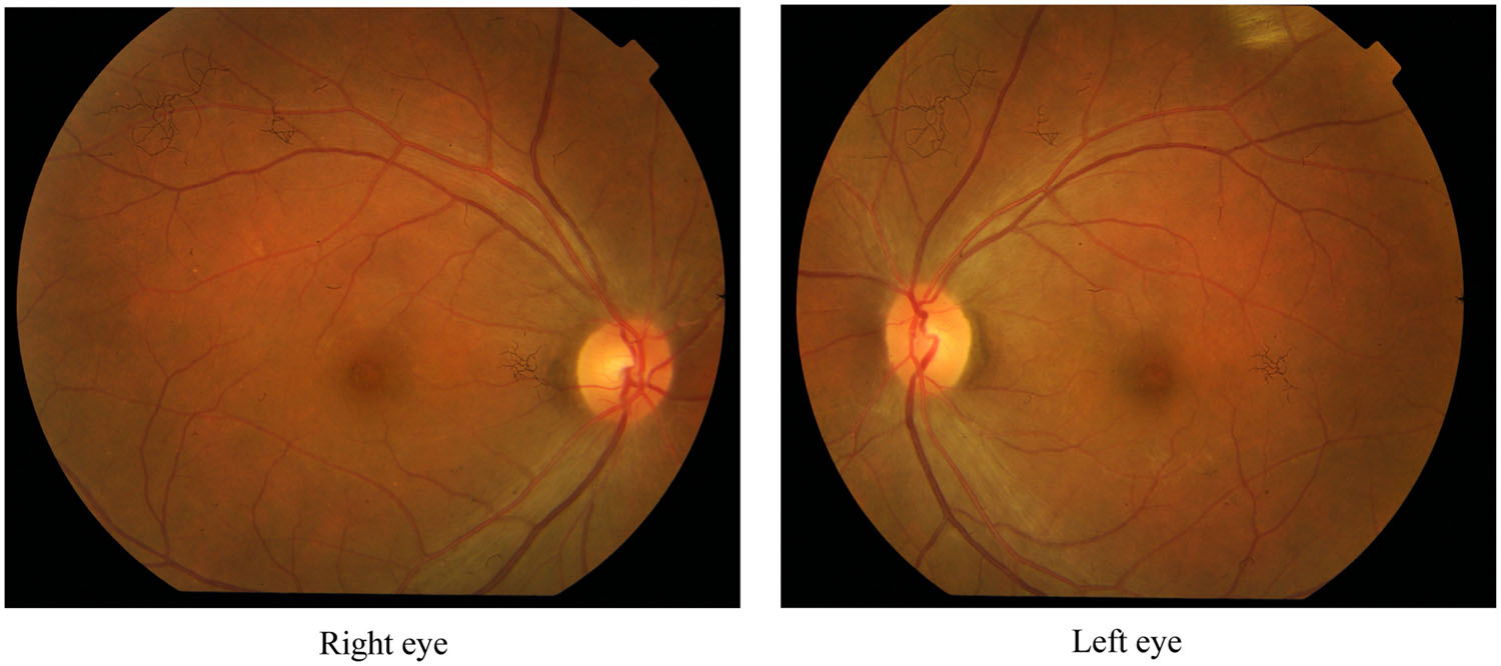
Posterior segment photo after treatment showing resolution of the optic disc swelling. Halo of disc edema and splinter haemorrhage resolved in both eyes.

**Figure 4:**
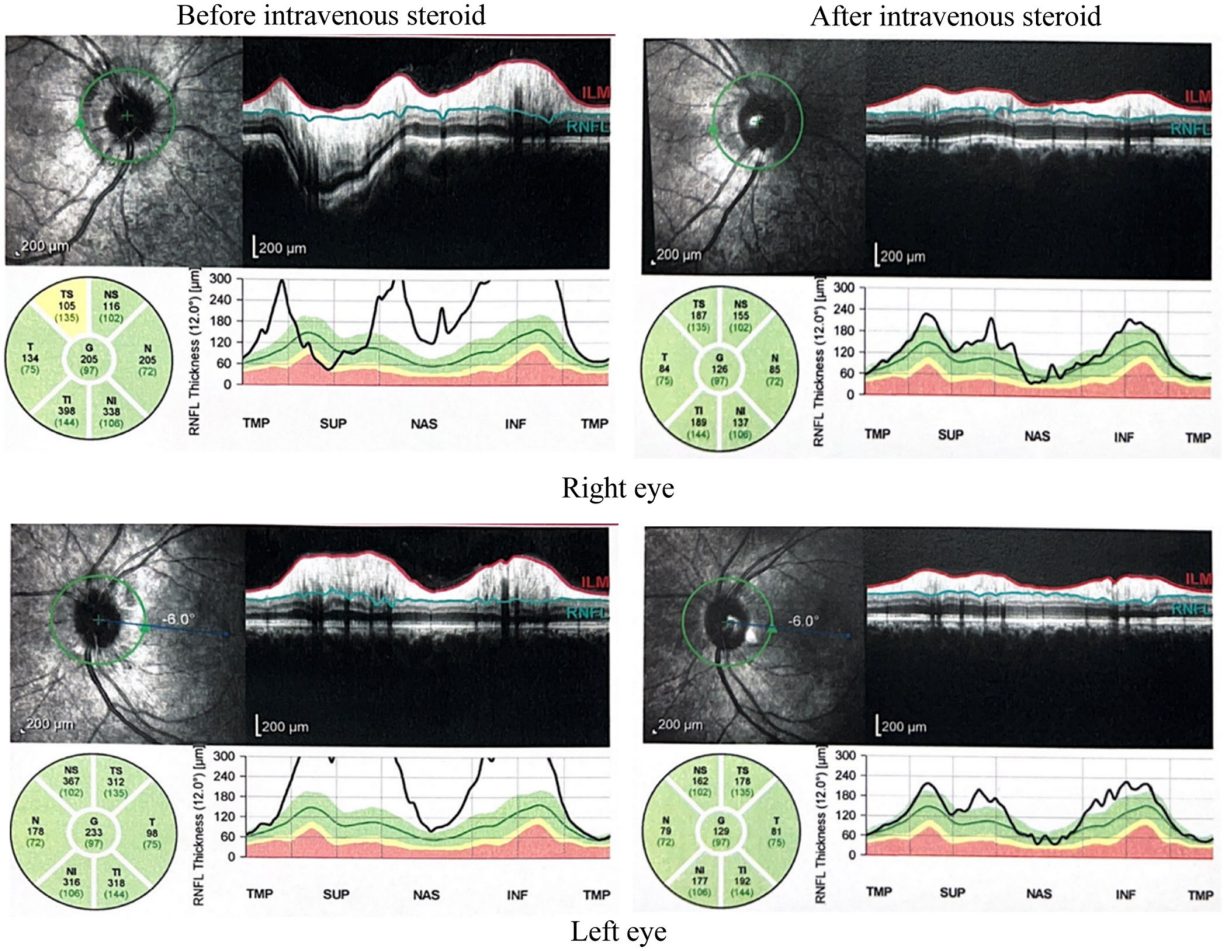
Optical coherence tomography of the peripapillary retina nerve fiber layer (RNFL) showing a swollen RNFL around the optic disc at initial presentation. Resolution was seen after intravenous steroid therapy.

A series of investigations were done to rule out the cause of optic neuritis. Serum MOG‐IgG indirect immunofluorescent antibody was significantly positive (titer of >1:10) with no detection of serum aquaporin‐4 antibody. Other infective and autoimmune screenings including HIV, VDRL, Hepatitis B and C, ANA, C3, and C4 were unremarkable. MRI of the brain and orbit with contrast revealed nonspecific multiple T2 weight/FLAIR hyperintense and T1 weight iso‐to‐hypointense white matter lesions at the subcortical regions of bilateral frontal lobes, left parietal lobe and left posterior cingulate gyrus. However, the optic nerves appeared normal without abnormal enhancement and thickening seen (Figure 2). Intravenous methylprednisolone 1 g per day was given for 3 days followed by oral prednisolone based on the Optic Neuritis Treatment Trial (ONTT) protocol. His vision improved markedly to 6/9 in both eyes with resolution of retrobulbar pain and optic disc swelling (Figures 3 and 4). The immunosuppression dosage of oral prednisolone was tapered and oral azathioprine was added. At 1 month, the disease was stable with no evidence of recurrence.

## 3 DISCUSSION

Classical MOG‐ IgG‐mediated demyelinating disease commonly presents with severe bilateral sequential visual loss associated with optic disc oedema, retinal venous congestion, optic perineuritis, and myelitis.^4^ This is a case of an adult male with subacute progressive MOG antibody‐positive bilateral optic neuritis which occurred a week after testing positive for COVID‐19 infection. The clinical features and course of the disease, in this case, appeared to be milder than that described in classical MOG‐IgG‐related optic neuritis.^4^

The pathophysiology of MOG optic neuritis is still incompletely understood. Theoretically, MOG‐IgG antibodies target the MOG receptors expressed on oligodendrocytes that serve as cellular receptors, adhesion molecules, or regulators of microtubule stability.^5^ These antibodies can circulate freely in the body. Following inflammation or infection, MOG antibodies can exhibit a pathological effect by gaining access to the central nervous system through disruption of the blood‐brain barrier.^6^ This access leads to the activation of the complement and immune‐mediated T cells, leading to the manifestation of various neurological autoimmune features including encephalitis, transverse myelitis, optic neuritis, cranial nerve palsy, nystagmus, visual field defect, and other autoimmune diseases such as Kawasaki, Miller Fisher, and Guillain–Barré syndrome.^7^, ^8^ Our case may suggest that one of the auto‐immune antibodies produced by this complex immune response could be the MOG‐IgG antibody.

Several cases of MOG‐IgG‐related optic neuritis after COVID‐19 infection have been reported. Zhou and colleagues reported a case of MOG‐IgG‐related optic neuritis with myelitis in a young adult male after a few days of COVID‐19 infection. The patient presented with bilateral, subacute sequential visual loss affecting the left eye followed by the right eye after 3 days associated with pain on eye movement, mild cough, and lower limb paraesthesia. Imaging showed bilateral enhanced optic nerves with myelitis of the central spinal cord. The patient had complete resolution of optic disc oedema after being given intravenous and oral steroid.^9^ Colantonio and colleagues also reported a case of MOG‐associated optic neuritis with myelitis who initially recovered from COVID‐19 infection but presented again after 2 weeks with altered mental status, paraesthesia, gait instability, and urinary incontinence. Supported with MRI of the brain and orbit, and lumbar puncture results, the patient was diagnosed with postinfectious COVID‐19 encephalomyelitis. Six months later the patient presented with left eye retro‐orbital pain and blurring of vision for 1 week. MRI brain and orbit revealed prechiasmatic enhancement of the left optic nerve, suggestive of optic neuritis. The MOG‐IgG antibody was positive and full recovery was achieved after treatment with intravenous and oral prednisolone.^10^

The spectrum of unilateral optic neuritis with MOG‐associated antibody post COVID‐19 infection have also been reported. Zoric and colleagues reported an elderly man with subacute right eye visual loss and headache. The disease presentation was right eye papillitis with positive MOG‐IgG antibody.^11^ Kogure and colleagues also described a 47‐year‐old male with acute left eye visual loss and eye pain 2 days after testing positive for COVID‐19 infection. The MRI revealed bilateral uniform enhancement of the optic nerve and its sheaths. High dose intravenous methylprednisolone given for three to 5 days, followed by a tapering dose of oral steroids were given in both patients, which resulted in good outcome with reduced eye pain and improved vision.^12^ A patient with a history of COVID‐19 pneumonia was also reported to develop unilateral optic neuritis 6 weeks later and responded well to pulse intravenous steroid therapy.^13^

The clinical characteristics, radiological findings, and dramatic treatment response to steroids firmly support an inflammatory disorder and are similar to the characteristic of MOG‐IgG‐mediated CNS disease. In our case, the patient presented with subacute ocular symptoms associated with mild COVID‐19 infection. There was clinical evidence of optic neuritis with nonspecific changes seen on the brain MRI. With significantly positive serum MOG levels, we diagnosed this patient as MOG‐related optic neuritis which occurred soon after a COVID‐19 infection. As illustrated in the previous reports, we also see a good immediate response in our patient with high‐dose intravenous corticosteroids.

Classical MOG‐related optic neuritis typically exhibits significant recurrence^14^ as opposed to COVID‐19 related neuritis which showed a lower recurrence rate, as illustrated in our case and other published cases. However, it is also likely that the occurrence of optic neuritis was an adverse effect of COVID‐19 vaccination. There have been reported cases of optic neuritis and other neurological deficits, such as demyelination, myasthenia gravis, Bell's palsy, and cranial nerve palsies, following the administration of vaccines.^15^ In our case, this pathological consequence may have occurred, as the patient had received a booster vaccine injection 2 months before the onset of the disease.

COVID‐19 infection causes a myriad of respiratory and neurological clinical manifestations from its early viral prodrome to the severe form. Even though immunological response is likely to occur in severe form of COVID‐19 infection, neurological manifestation has also been reported in mild cases of COVID‐19 infection, as shown in our patient.

## 4 CONCLUSION

COVID‐19 infections provide new challenges as they may result in unexpected immunological responses. The pathogenesis of MOG‐related CNS inflammation after COVID‐19 infections is still unclear. However, a good response is seen with high‐dose corticosteroid therapy, given in a longer tapering dose.

## AUTHOR CONTRIBUTIONS

**Mohamad Azlan Zaini**: Writing—original draft. **Ayesha Mohd Zain**: Project administration; validation; writing—review & editing. **Norshamsiah Md Din**: Project administration; supervision; validation; writing—review & editing. **Chenshen Lam**: Supervision; writing—review & editing.

## Supporting information

Supporting information.
